# Perturbations at the ribosomal genes loci are at the centre of cellular dysfunction and human disease

**DOI:** 10.1186/2045-3701-4-43

**Published:** 2014-08-19

**Authors:** Jeannine Diesch, Ross D Hannan, Elaine Sanij

**Affiliations:** 1 Growth Control Laboratory, Research Division, Peter MacCallum Cancer Centre, St. Andrews Place, East Melbourne, Victoria 3002, Australia; 2 Sir Peter MacCallum Department of Oncology, University of Melbourne, Parkville, Victoria 3010, Australia; 3 Department of Biochemistry and Molecular Biology, Monash University, Clayton, Victoria 3800, Australia; 4 Department of Biochemistry and Molecular Biology, University of Melbourne, Parkville, Victoria 3010, Australia; 5 Division of Cancer Medicine, Peter MacCallum Cancer Centre, St. Andrews Place, East Melbourne, Victoria 3002, Australia; 6 School of Biomedical Sciences, University of Queensland, Brisbane, Queensland 4072, Australia; 7 Department of Pathology, University of Melbourne, Parkville, Victoria 3010, Australia

**Keywords:** rDNA, RNA Polymerase I, Nucleoli, UBF

## Abstract

Ribosomal RNA (rRNA) gene (rDNA) transcription by RNA Polymerase I (Pol I) drives cell growth and underlies nucleolar structure and function, indirectly coordinating many fundamental cellular processes. The importance of keeping rDNA transcription under tight control is reflected by the fact that deranged Pol I transcription is a feature of cancer and other human disorders. In this review, we discuss multiple aspects of rDNA function including the relationship between Pol I transcription and proliferative capacity, the role of Pol I transcription in mediating nucleolar structure and integrity, and rDNA/nucleolar interactions with the genome and their influence on heterochromatin and global genome stability. Furthermore, we discuss how perturbations in the structure of the rDNA loci might contribute to human disease, in some cases independent of effects on ribosome biogenesis.

## Introduction to rDNA transcription by Pol I

In human cells 300 copies of the rRNA genes are arranged in repeated arrays located in nucleolar organizer regions (NORs) on the short arms of the acrocentric chromosomes [1-5]. Pol I transcribes rDNA to produce the 47S rRNA, which is the precursor of the mature 28S, 5.8S, and 18S rRNAs. Together with the 5S rRNA transcribed by Pol III in the nucleoplasm, these rRNAs form the nucleic acid backbone of the ribosome. The other major components of the ribosome are the ~78 ribosomal proteins (RPs), whose genes are transcribed by Pol II, and are assembled with the rRNAs to form functional ribosomes [6]. In growing mammalian cells, rRNA synthesis by Pol I accounts for 35- 60% of all nuclear transcription while rRNA represents nearly 80% of the steady-state cellular RNA content [7,8]. Accordingly, Pol I transcription rate is tightly coupled to cellular growth and proliferation rates, and is modulated in response to a multitude of cellular cues including nutrient availability, growth factor signaling, cell cycle progression, differentiation, senescence, and DNA damage [8-13]. Inhibition of Pol I transcription leads to cell cycle arrest associated with apoptosis, senescence or autophagy depending on the cell type [14-17]. Hence, rDNA transcription has been proposed to directly couple cell growth to cell cycle progression and to influence the decision of a cell to arrest in response to various forms of stress [18,19]. Not surprisingly, it is becoming increasingly clear that dysregulation of Pol I transcription is linked to the aetiology of a broad range of human diseases [20].

rDNA transcription underpins the structure of the nucleoli, which form around active clusters of rDNA [21]. However, the primary function of the nucleoli is not limited to the production of the ribosomal subunits [22-25]. Bioinformatic analysis of the nucleolar proteome revealed that only 30% of the nucleolar proteins are involved in ribosome biogenesis, while included in the rest are factors associated with mRNA metabolism, chromatin structure, cell cycle control, DNA replication and repair [21,22,26-31]. The nucleolus indirectly, through sequestration and release of these proteins, has the ability to modulate a diverse range of cellular functions including regulating tumor suppressor and proto-oncogene activities, cell-cycle control, DNA replication and repair, and stress signaling independent of ribosome biogenesis [23,25,26,32-41]. Perturbation of nucleolar structure and function leads to a response termed “nucleolar stress”, characterized by the accumulation of the tumour suppressor protein p53 leading to induction of apoptosis, senescence or cell cycle arrest [18,23,42-50]. Therefore, the nucleolus is at the centre of coordinating rDNA transcription, ribosome subunit biogenesis, cell cycle progression and cellular stress responses [17,40,45,48].

Recent evidence also suggests that the epigenetic status of the rDNA repeats and the integrity of the nucleolus can modulate cellular homeostasis beyond ribosome biogenesis and nucleolar stress. Spatial organization of the genome around the nucleoli and the interactions of specific chromatin domains with the nucleoli are both suggested to affect the various functions of the nucleoli and *vice versa*[51,52]. Furthermore, the repetitive nature and high transcription rates of the rRNA genes render the rDNA some of the most fragile sites in the genome [53]. Somewhat surprisingly, only a fraction of the rRNA genes is transcriptionally active at any given time. In yeast, the silent rDNA copies appear to play an essential role in maintaining the genetic stability of the rDNA repeats [54]. Epigenetic silencing of rDNA has also been proposed to mediate nucleolar integrity, genomic stability, and the global regulation of gene expression [52,55], with these having downstream effects on cellular processes such as senescence and aging [11,56-58].

This review provides an overview of the mechanisms that regulate rDNA transcription. We will discuss the spatial organization of the nucleoli around actively transcribed rDNA and their potential functional interactions with the rest of the genome, and the notion of rDNA instability promoting genome-wide instability and influencing cellular functions such as, maintainance of heterochromatin, DNA damage response and aging. Further, we present our current knowledge of human diseases specifically associated with deregulated Pol I transcription.

### Regulation of Pol I transcription

In addition to RNA Polymerase I, optimal rRNA gene transcription requires a number of accessory factors that facilitate Pol I recruitment, initiation, promoter escape, elongation, termination and re-initiation [12,59,60]. Pol I transcription begins with the formation of the preinitiation complex (PIC) by the upstream binding factor (UBF) and the TBP-containing complex selectively factor (SL-1, also called TIF-1B) at the rDNA promoter. SL-1 confers promoter sequence specificity by recognizing the core promoter element and it promotes a stable interaction between UBF and the rDNA promoter [61-64]. In turn, UBF binds the upstream and core promoter elements (UCE and CORE) as a dimer, possibly looping the intervening DNA into a nucleosome like structure termed the enhancesome, which brings the activating UCE sequence into close proximity with the core promoter element [65-67]. UBF binding also promotes stabilization of SL-1 interaction with the rDNA promoter [68]. The resultant UBF/SL-1 complex then facilitates recruitment of an initiation-competent subpopulation of Pol I, defined by the presence of the basal regulatory factor RRN3 (also called TIF-1A), to form a productive PIC at the rDNA promoters [8,68-72]. Furthermore, UBF interacts with the entire transcribed region [73-75], not just promoter elements, and can regulate promoter escape [76] and Pol I elongation in response to growth stimuli [77].

Despite the high demand for rRNA synthesis, only a subset of rRNA genes is transcribed at any given time. In mammalian cells, rDNA chromatin can exist in at least four distinct states [78,79]. The first two states are defined as open/accessible chromatin structures. They are bound by UBF, which is essential in determining and maintaining the active rDNA state [80], and are characterized by being transcriptionally active and transcriptionally poised states. RNA interference-mediated depletion of UBF silences active rRNA genes by promoting histone H1-induced assembly of closed transcriptionally inactive chromatin [80]. The two states of inactive rDNA chromatin represent silenced rDNA that is devoid of UBF and Pol I and are distinguished by the presence or absence of methylated CpG dinucleotides in the rDNA promoter. The silenced non-methylated fraction is thought to carry activating histone marks that can presumably transit to the open chromatin state if rDNA becomes bound by UBF. In contrast, the methylated rDNA population is believed to carry repressive histone marks and is established and stably maintained by the nucleolar repressive complex NoRC, a member of the ATP-dependent chromatin remodeling complexes [81-83]. Studies in yeast suggest that these silenced rRNA genes are required for efficient DNA recombination repair and thus play an important role in maintaining rDNA stability [56].

### Organization of the nucleoli

The nucleolus is the subnuclear site of ribosome biogenesis and its formation around active NORs requires ongoing rDNA transcription. The structure of nucleoli is highly dynamic and is tightly coordinated with cell cycle progression. Nucleoli disassemble at the onset of mitosis coinciding with inactivation of Pol I transcription and reassemble during telophase as rDNA transcription is reinitiated. The rate of rRNA gene transcription reaches a maximum in S- and G2 phases, is halted at the onset of mitosis and slowly reactivated as cell enter G1 [84]. Cell cycle mediated regulation of rDNA transcription is facilitated by post-translational modifications of components of the Pol I transcription machinery and its associated transcription factors such as phosphorylation of UBF and SL-1 [85-87]. Interestingly, few components of the Pol I machinery, including Pol I as well as UBF and SL-1 remain associated with active NORs throughout mitosis, presumably to allow reactivation of Pol I transcription upon entry into the G1 phase [88-91]. Formation of nucleoli requires association of UBF with the rDNA, which acts as a scaffold to initiate and maintain nucleolar competency even in absence of rDNA transcription [91-93].

Once established, the nucleolus comprises three major structural and functional subcompartments defined by their texture and contrast using electron microscopy, the fibrillar centre (FC), the dense fibrillar component (DFC) and the granular component (GC) [21,24,94,95]. The FCs, in which the NORs can be found, are clear areas surrounded by highly contrasted DFCs. Depending on the rate of ribosome biogenesis one nucleolus can consist of several FCs whilst exponentially growing cells can exhibit several large nucleoli [96]. The majority of the 47S precursor rRNA is thought to be synthesized at the boundary between the FC and the DFC [97]. DFCs harbour the small nucleolar ribonucleoproteins (RNPs) necessary for the first steps of rRNA processing, whereas the late steps of rRNA processing and assembly of the small (40S) and large (60S) ribosome subunits take place in the GCs [21,24].

The organization, size and protein composition of the nucleoli change dramatically during the cell cycle and under different cellular conditions, including stress and viral infections [39,40,48,98-102]. Over 4500 proteins reside within human nucleoli and through the control of their sequestration and release, nucleoli modulate a diverse range of cellular functions such as control of the cell-cycle apparatus, ageing, cellular stress responses, mRNA export and modification, protein degradation, assembly and export of RNPs [21-28,31,33,45,48,103-105]. One such sequestration function involves non-coding RNA produced from the intergenic spacer (IGS), which separates the rDNA repeats. This noncoding RNA is produced in response to various stimuli including acidosis, heat shock and transcriptional stress and is capable of capturing and immobilizing key cellular proteins that encode a discrete peptidic code referred to as the nucleolar detention sequence (NoDS) [32]. Disruption of the NoDS/intergenic RNA interaction enables proteins to escape nucleolar sequestration and retain their nucleoplasmic function [32,106]. NoDS-carrying proteins are involved in diverse functions including ubiquitination, proteasomal degradation, protein folding, DNA replication and methylation [107]. Nucleolar retention of proteins away from their normal sites of action is a further example of the multifunctional nature of the nucleoli [33,45,48,108].

### Nucleolar coordination of cellular stress response

Nucleolar integrity is tightly linked to rRNA gene transcription and ribosome biogenesis. Downregulation of Pol I transcription seems to be a major strategy to maintain cellular homeostasis under adverse growth conditions or metabolic deficits [83,87,109-113]. Furthermore, a variety of abnormal metabolic conditions, cytotoxic agents, and physical insults induce alterations in nucleolar structure and function, and ribosome biogenesis [48,110,114]. The rate of ribosome biogenesis is now thought to function as a highly sensitive cellular sensor of stress.

Virtually any major cellular perturbation that uncouples the processes driving ribosome synthesis and assembly results in the activation of a regulated series of events that are controlled through the nucleoli, which activate cell cycle checkpoints leading to cell cycle arrest or cell death [18,44,46-48,115-117]. This process has been termed nucleolar stress or ribosome surveillance. One of most prominent events downstream of nucleolar stress is activation of the p53 tumour suppressor protein, a key regulator of stress-induced apoptosis, DNA repair, cell cycle arrest, and senescence [18,22,42-44,46,48,50,118,119]. In this pathway p53 is activated by the essential 60S ribosomal proteins RPL11 and RPL5 that function in a MDM2 inhibitory complex with 5S rRNA, which binds MDM2 and blocks its function to degrade p53 [120-129]. Thus, a dynamic equilibrium exists in the cell, which couples on-going ribosome biogenesis to p53 protein stability, such that the RPL5/RPL11/5S rRNA complex is either incorporated into nascent ribosomes or is bound to MDM2. Therefore, this is a key pathway that enables the coordination of ribosome production with cell proliferation [120]. We and others have also identified p53 independent checkpoints that are activated in response to inactivation of Pol I transcription, although the mechanism controlling this process is not understood (Quin J. and Sanij E. unpublished data), [130,131]. Taken together, the direct coupling of Pol I transcription and the ribosome biogenesis rate through the nucleolar stress pathway ensures a coordinated response to a variety of proliferative and stress stimuli.

### Genome organization around the nucleoli

The structure of the genome is highly dynamic and is closely coupled with gene regulation. It is now well accepted that the nucleus is organized into chromosome territories and transcription factories in which functionally related genes cluster together allowing their concerted regulation [132]. Furthermore, the spatial organization and location of chromosomes and their interactions with other nuclear substructures ensures that transcription is correctly regulated and maintains genome stability [133,134]. The discovery of structural and functional links between the nucleolus and the rest of the genome have led to the proposal that the nucleolus plays a key role in mediating nuclear architecture [135].

The periphery of the nucleolus contains satellite DNA repeats, which are thought to be involved in the formation of perinucleolar heterochromatic domains surrounding the nucleolus as a dense shell [136]. CpG-methylated silent rDNA assembles in proximity to the perinucleolar heterochromatin, suggesting a specific relationship between these heterochromatic regions and silent rDNA copies [137]. Indeed, NoRC mediated silencing of rDNA is important for the formation of the perinucleolar domains [57]. Regions found in the perinucleolar region, termed nucleolar-associated domains (NADs), include centromeres of chromosome 1 and 9 and chromosome Y heterochromatin [138]. Recent high-resolution genome-wide mapping of NADs revealed the association of nucleoli with various satellite repeats (mainly alpha-, beta- and (GAATG)_n_/(CATTC)_n_-types) and genes belonging to the zinc finger, olfactory receptor and immunoglobulin gene families [139]. In addition, the region flanking the rDNA on the telomeric side contains a large tract of a satellite repeat family that is specific to the acrocentric chromosomes [140]. Similar to lamina-associated domains (LADs), which are localized at the nuclear envelope, a strong correlation of NADs with AT-rich sequences and regions with low gene density has also been observed [141].

Genes enriched in the NADs are characterized by repressive histone marks and a lack of gene expression. Indeed, the NADs have been proposed to serve as a distinct nuclear space with a primary function in maintaining repressive chromatin states [51,141]. For example, the inactive X-chromosome visits the nucleoli during S-phase to maintain its repressive state [142]. A repressive, inhibitory effect of the nucleoli on gene expression has also been demonstrated by inducing nucleolar association in response to random multicopy insertion of ectopic 5S rDNA sequences in ES cell lines, which resulted in transcriptional repression of genes adjacent to the insertion site [143]. Indeed, RNA genes transcribed by RNA polymerase III, such as 5S rRNA, tRNA and U6 snRNA are also localized within NADs and are thought to recruit adjacent protein coding genes to the nucleoli [144,145]. As Pol III-transcribed genes and derived sequences make up a large proportion of the genome, it has been proposed that these can significantly contribute to nucleolar association of neighboring genes for the purpose of mediating gene silencing [143]. However, there is no enrichment of these elements in the regions immediately flanking the rDNA arrays even though they show perinucleolar localization [140]. Apart from Pol III-transcribed genes, several other factors have been suggested to tether chromatin regions to the nucleoli. One example is the CCCTC-binding factor (CTCF), which regulates various cellular processes and has recently been shown to affect nuclear structure by binding to insulator sequences, preventing crosstalk between neighboring sequences, and facilitating chromatin loops between CTCF binding elements [146]. CTCF has been shown to localize to the nucleoli where it interacts with nucleolar proteins such as nucleophosmin [147] and UBF [148]. A third class of potential nucleolus-tethering factors is long noncoding RNAs (lncRNAs). For instance, the lncRNAs Kcnq10t1 promotes the lineage-specific inhibition of genes in the Kcnq1 domain by inducing their relocation to the nucleoli [149,150]. A similar mechanism has been shown to be involved in the perinucleolar targeting of the inactive X chromosome mediated by the Xist RNA [142]. Further, unique lncRNAs are produced from the rDNA arrays flanking regions, although their function has not been determined [140].

Taken together, the observed enrichment of heterochromatic regions and transcriptionally repressed genes in perinucleolar domain suggests that the NADs represent a novel mechanism of gene silencing. However, more experiments need to be performed to completely rule out the possibility of nucleolar localization being a mere consequence of inactive gene expression. Additionally, the composition of NADs in disease and their biological relevance are not well understood.

### rDNA stability maintains genome integrity

The repetitive nature of the rDNA leaves them vulnerable to loss or gain of rDNA copies through a high rate of recombination [151,152], although this remains poorly studied in humans [2]. Changes in the number of rDNA copies can create an imbalance in the ratio of silent to active repeats that affects global heterochromatin content [57]. This in turn can lead to deregulated gene expression, promoting genome instability [153]. Indeed, rDNA instability has been observed in Bloom syndrome patients, which harbour a mutation in the DNA helicase BLM gene leading to dysregulated homologous recombination (HR) [154]. rDNA instability can also be detected in cells deficient for ataxia-telangiectasia (ATM), the primary sensor of DNA double stranded breaks. In Bloom syndrome and ataxia-telangiectasia patients, rDNA instability correlates with increased cancer predisposition [154]. The rDNA is a recombinational hotspot in cancer, suggesting that rDNA instability may be a mechanism of global genomic instability and could drive the etiology and progression of cancer [53,155]. Neurodegeneration also appears to be associated with instability of rDNA [156], including Alzheimer’s disease [157].

The importance of rDNA copy number and rDNA integrity is implied by the existence of a well-regulated maintenance system in yeast which keeps rDNA copy number at a uniform level, as well as the tight regulation of rDNA recombination [152]. During yeast HR, which is the major cause of rDNA copy variation [158-160], rDNA repeats that are being repaired are transported out of the nucleolus into nuclear repair foci, presumably to prevent rDNA hyper-recombination. This nucleolar exclusion is mediated by the Smc5-Smc6 complex and the sumoylation of the central HR protein Rad52 [161]. In addition, anti-recombinatoric regulators such as Srs2 are found in close proximity to the rDNA [161].

Several studies in yeast suggest a strong correlation between rDNA copy number and genome integrity [152,162]. In 2008, Kobayashi T. (National Institute of Genetics, Japan) introduced the “rDNA theory” of aging, in which rDNA instability-dependent aging is proposed to induce senescence and prevent global genome instability [162,163]. In *Drosophila*, general heterochromatin content appears to be directly influenced by the number of rDNA copies suggesting that rDNA instability may affect heterochromatin maintenance [58]. Consistent with this, induced rDNA deletions altered the expression of hundreds to thousands of euchromatic genes throughout the genome [55]. The relationship between rDNA and genome stability has also been demonstrated in mouse cells, where loss of rDNA silencing results in destabilization of the perinucleolar heterochromatin, which is crucial for ensuring genome stability [57,83].

**Figure 1:**
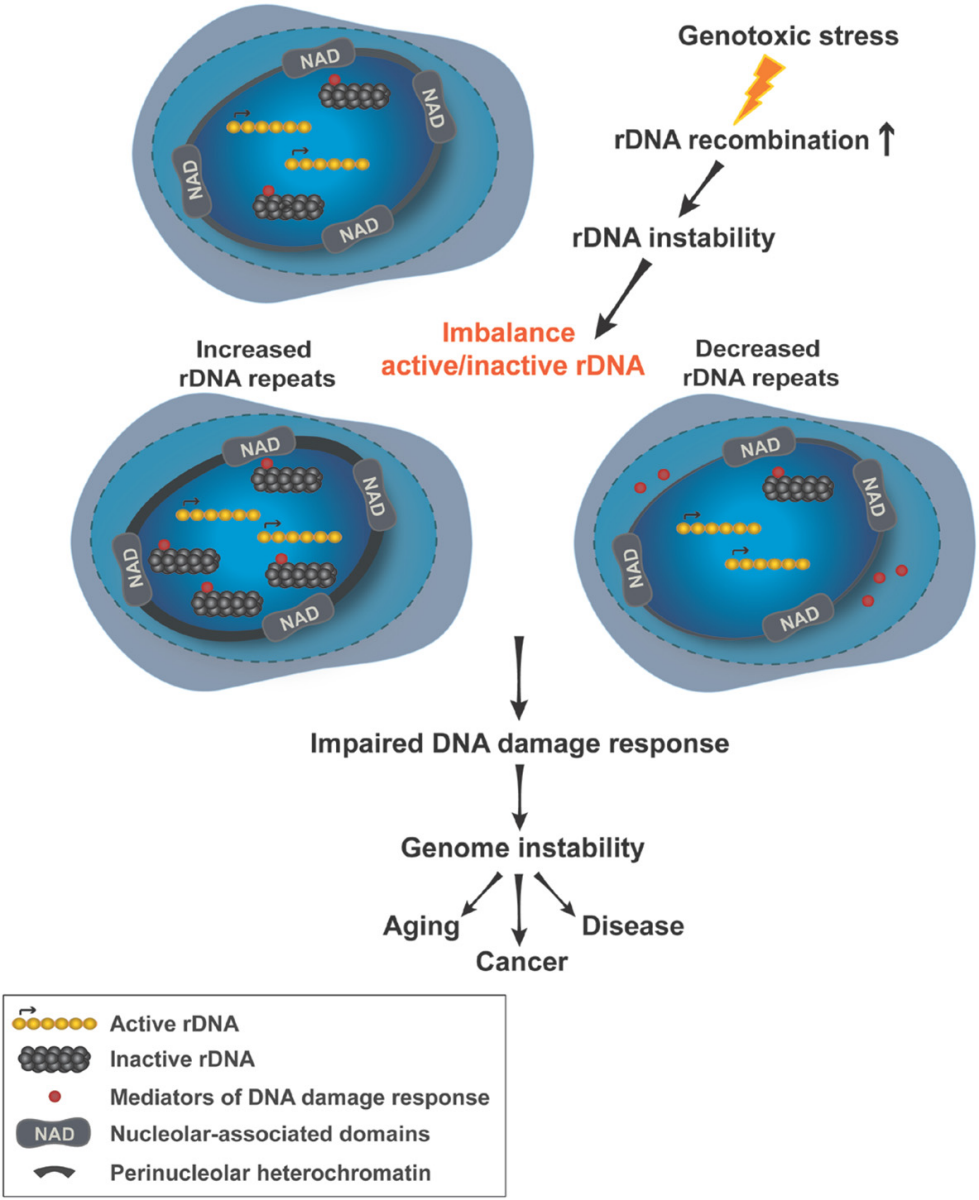
**Proposed model of genome-wide consequences of rDNA instability.** Under normal conditions rDNA copy number is maintained at uniform levels due to tight control of homologous recombination (HR) at rDNA loci. Induced HR in response to DNA damaging stimuli can lead to an increase or decrease in rDNA copy number resulting in an imbalance in the ratio of active to silent rDNA. This, in turn, will affect ribosome biogenesis independent functions of rDNA, which include regulation of heterochromatin content and DNA damage response leading to global genome instability and promoting aging or disease.

In addition to the role of rDNA in regulating heterochromatin, silent rDNA repeats are required for DNA damage repair, an essential pathway for preventing genome instability. The influence of rDNA on DNA damage has been shown utilizing yeast strains that have reduced rDNA copy numbers, in which most or all copies are actively transcribed [56,164]. These low-copy strains have impaired DNA damage repair during S-phase and consequently higher sensitivity to DNA damaging agents such as ultraviolet radiation and methyl methanesulfonate [56]. The sensitivity to DNA damage is due to the inability of condensin, which is required for sister-chromatid cohesion and facilitates DNA repair, to bind the active rDNA repeats [56]. A relationship between condensin and rDNA has been demonstrated in several other studies further supporting the idea that silent rDNA repeats are required for rDNA repair [165-168]. Moreover, studies in yeast led to the proposition that silent copies of rDNA can sequester mediators of the DNA damage repair pathway and that the rDNA acts as a stress centre for DNA damage [56,158]. Hence, variations in rDNA copy number may influence cellular responses to DNA damage (Figure 1).In summary, rDNA repeats are crucial players in the maintenance of genome stability. Perturbations at the rDNA loci resulting in either a decrease or increase in rDNA copies have a great impact on cellular processes, including heterochromatin structure and function, global gene expression and DNA damage response. These processes can then, in turn, promote aging, cancer and disease (Figure 1).

### Deregulation of rDNA transcription in disease

Upregulation of Pol I activity is common in diseases associated with profound changes in cellular growth, such as cardiac disease [169] and cancer [15-17,116,170,171]. Indeed, enlarged nucleoli indicative of increased Pol I transcriptional activity and ribosome biogenesis are associated with cardiac pathophysiology [169]. Similarly, the nucleoli of cancer cells are enlarged and increased in number, and thus the morphology of nucleoli is commonly used as a diagnostic marker for transformed cells clinically corresponding to adverse prognosis [172,173]. These data suggest it is incorrect to consider rDNA transcription as simply a “house keeping” process that reflects the metabolic state of somatic cells. Indeed, recent findings demonstrate that deregulation of Pol I transcription is necessary for malignant transformation in certain systems [174].

Pol I activity is frequently deregulated in cancers by oncogene activation and/or tumor suppressor inactivation [17,18,20] and this has been previously explained in terms of an increased demand for ribosomes in highly proliferative cells. Certain tumour cells however appear to be very susceptible to inhibition of Pol I transcription leading to induction of cancer cell death [14,174,175]. This therapeutic benefit is due to tumour-specific induction of the nucleolar stress pathway leading to activation of p53 and cell death by apoptosis. Activation of the p53 pathway following inhibition of Pol I transcription in tumour cells is not a consequence of ribosome insufficiency, suggesting that the cells are responding directly to perturbations in rDNA transcription [174]. The therapeutic efficacy of targeting deregulated Pol I activity in cancer is currently being investigated using a selective small molecule inhibitor of Pol I transcription, CX-5461 [14,174], that is in phase 1 clinical trials (Peter MacCallum Cancer Centre, Melbourne, Australia) as a mechanism to selectively and non genotoxically activate the p53 pathway in cancer cells.

Further, deregulation of rDNA transcription contributes to the pathology of several rare human genetic disorders (reviewed in [20]). These conditions are caused by loss of function mutations in factors directly associated with Pol I transcription, modulators that impact on Pol I transcription, or mutations that affect rRNA processing or rRNA modifications. These diseases, as well as those associated with loss of function mutations in the molecular constituents of the ribosome, are termed ribosomopathies (reviewed in [20,176]). To date at least six human syndromes (Treacher Collins syndrome, Blooms and Werner syndrome, Cockayne Syndrome, Siderius X-linked mental retardation, and a group of Filamin A associated diseases) are associated with mutations in genes whose products interact directly with rDNA or the Pol I transcriptional complex. Generally, these mutations are associated with reduced rRNA synthesis and nucleolar malfunction [20].

Cohesinopathies are another group of human congenital disorders associated with deregulated Pol I transcription. Cornelia de Lange and Roberts Syndrome are associated with mutations in genes encoding either regulators or structural components of the cohesin complex, which is critical for sister chromatid cohesion, chromosome segregation during S phase, chromosome condensation, DNA damage repair and gene regulation including Pol I transcription of the rRNA [18,177,178]. Although the cohesin complex has been reported to interact with rDNA [178], the molecular mechanisms that lead to deregulation of Pol I transcription in these diseases have not been established. While ribosomopathies and cohesinopathies are unique, collectively they exhibit overlapping symptoms including craniofacial abnormalities and growth retardation. Downregulation of rDNA transcription has been proposed to determine cell fate and to act as a trigger for cellular differentiation [179,180]. Thus, it is plausible that deregulation of Pol I transcription is the underlying cause of these common features [20].

Downregulation of rRNA synthesis and nucleolar size has also been observed during aging [181,182]. Altered rRNA gene transcription and disruption of nucleolar integrity and function are associated with the pathogenesis of age-related neurological disorders such as Alzheimer’s disease [157], Huntington’s diseases [183,184], Parkinson disease [185] and spinocerebellar ataxias [186]. More recently, elevated rDNA copy number was detected in patients with dementia with Lewy bodies, which involves neurodegeneration of the cerebral cortex [156]. Moreover, increased genomic content of the 18S rDNA region and an increase in rDNA silencing, distinguished by rDNA promoter methylation has been detected in the parietal cortex of Alzheimer’s disease patients [157].

The evolving paradigm of the nucleolus being a key regulator of cellular homeostasis implicates nucleolar stress resulting from deregulation of rDNA transcription in the development of these diseases. Unquestionably, further mechanistic investigations are required in order to examine how perturbations of rDNA stability and function, independent of ribosome biogenesis and nucleolar stress, are involved in the aetiology of these diseases.

## Conclusions

Taken together, this review advocates a role for the nucleoli in genome organization and the regulation of gene expression beyond its classic role in ribosome biogenesis and nucleolar stress response. Variation in rDNA copy number alters the ratio of active to silent rDNA repeats, which in turn can alter heterochromatin content. As such, changes at rDNA loci could affect gene expression and alter global genomic stability driving an imbalance in cellular homeostasis leading to disease. Analysis of rDNA/nucleolus interactions with chromatin domains concomitant with genome-wide gene expression analyses under various cellular conditions are the next steps necessary to understand rDNA/nucleolar functions at genome-wide as well as system levels.

## Abbreviations

ATM: CCTF, CCCTC-binding factor; DFC: Dense fibrillar component; FC: Fibrillar centre; GC: Granular component; HR: Homologous recombination; IGS: Intergenic spacer; LADs: Lamina associated domains; lncRNA: Long noncoding RNA; NADs: Nucleolar associated domains; NOR: Nucleolar organizer region; NoRC: Nucleolar repressive complex; NoDS: Nucleolar detention sequence; PIC: Preinitiation complex; Pol: RNA polymerase I; rDNA: Ribosomal gene; RNPs: Ribonucleoproteins; RP: Ribosomal proteins; rRNA: Ribosomal RNA; SL-I: Selectivity factor 1; UBF: Upstream binding factor.

## Competing interests

The authors declare that they have no competing interests.

## Authors’ contributions

JD and ES contributed equally to writing this review. RDH provided substantive intellectual input. All authors read and approved the final manuscript.

## Authors’ information

ES received her PhD from Monash University (Melbourne, Australia) in 2003 and was awarded a Cancer Research UK Postdoctoral Fellowship to undertake research in Dr. Gordon Peters laboratory at the London Research Institute (London, United Kingdom). Since 2006, she worked as a Senior Research Scientist in the Growth Control Laboratory at the Peter MacCallum Cancer Institute (Melbourne, Australia). Her studies examine epigenetic regulation of ribosomal gene (rDNA) transcription (Sanij et al., JCB 2008) with a focus on the use of next generation sequencing to characterize the chromatin state of the rDNA. Further, she made seminal contributions in identifying deregulation of Pol I transcription as a requirement for malignant transformation and the therapeutic efficacy of targeting Pol I transcription in cancer (Drygin et al., Cancer Res 2011; Bywater et al., Cancer Cell 2012; Hein et al., Trends Mol Med 2013).
